# A New Dimension to Relative Age Effects: Constant Year Effects in German Youth Handball

**DOI:** 10.1371/journal.pone.0060336

**Published:** 2013-04-26

**Authors:** Jörg Schorer, Nick Wattie, Joseph R. Baker

**Affiliations:** 1 Institute of Sport and Exercise Sciences, University of Muenster, Münster, Germany; 2 School of Kinesiology and Health Science, York University, Toronto, Canada; Universidad Europea de Madrid, Spain

## Abstract

In this manuscript we argue for a broader use of the term ‘relative age effect’ due to the influence of varying development policies on the development of sport expertise. Two studies are presented on basis of data from Schorer, et al. [1]. The first showed clear ‘constant year effects’ in the German handball talent development system. A shift in year groupings for the female athletes resulted in a clear shift of birth year patterns. In the second study we investigated whether the constant year effect in the national talent development system carried over to professional handball. No patterns were observable. Together both studies show that a differentiation of varying effects that often happen simultaneously is necessary to understand the secondary mechanisms behind the development of sport expertise.

## General Introduction

Birthdates can play an important role in human development. In sport, placement into teams is commonly based on the chronological age of members of a cohort. Dividing young athletes into age-groups is intended to reduce maturational differences between athletes during their childhood and adolescence thereby allowing more balanced coaching and evaluation; however, employing age-cohorts seems to provide participation and attainment disadvantages to some members [2], [3]. For example, in a system using January 1st as a cut-off date to group young athletes, an individual born close to the selection date is almost one year older than a cohort-member born at the end of August. The interaction between annual age-groupings and the individual characteristics (e.g. chronological age compared to peers) of athletes results in *relative age effects* (RAEs) [4].

Several studies show RAEs across a range of sports, including baseball [5], basketball [6], soccer [7]–[13], handball [1], [14], [15], ice-hockey [16], rugby [17] and swimming [18]. Several interacting mechanisms have been proposed to explain RAEs. There is some evidence that highly competitive talent identification systems provide advantages to the relatively older athletes and disadvantages to their younger peers [19]. This maturational hypothesis is based on the supposition that athletes born close to the selection date benefit from their advanced physical and cognitive maturation [20], [21]. In sports requiring power and speed, greater height and body mass might underline a higher chance of success and good performance. As a consequence, coaches confuse maturation for talent thereby leading to an increased likelihood that relatively older youths are identified as being talented and selected for higher levels of competition [22]. There is some evidence that this mechanism is a cause of RAEs [23], [24] and therefore researchers have focused on the maturation-selection hypothesis when considering the consequences of being selected (or equally important, not selected) for the next level of development [1]. Helsen, Starkes, and Van Winckel [25] demonstrated that being selected for talent development opportunities is a critical factor in promoting skill acquisition and improved performance. Selected older members of a cohort subsequently get access to better resources such as practice facilities, coaching and competition and therefore have a higher chance of reaching elite professional status [2]. Those not selected are at a disadvantage since they have less access to performance resources that might help them to compensate for the maturational differences that are most profound at early stages of an athlete's development [1].

Interestingly, diversity exists in the age-based and selection date-based policies that are used to group individuals for sport participation, and this diversity creates opportunities for additional research to understand these effects. One opportunity for research stemmed from the structure of Masters athlete participation, where participation is grouped into five-year age bands (e.g., 40–44, 45–49, 50–54 years). Research suggests that participant's ages within their current age band (described as ‘relative age’ since it relates to one's age relative to others in their age band) influences the likelihood of both participation and success [26], [27]. More specifically, those in the early stages of their age band (e.g., those aged 40 and 41 years in a 40–44 years age band) had both higher participation rates and likelihoods of success.

However, the use of the same term (i.e., relative age) in the instance of Master's athlete research *and* previous relative age research, when referring to two distinct effects, is clearly problematic. Wattie, Cobley, and Baker [28] suggested that a new term, *constituent year effects*, be used for the type of effects observed between multiple cohorts within multiyear age bands. Of relevance to the current study, Wattie et al [28] observed that these two phenomena (relative age and constituent year effects) can be observed in the same context/sample [29], [30]. For example, youth ice hockey participation in Canada uses both annual age grouping policies and two-year age bands. Therefore, youth in this context have a relative age *and* a constituent year. Players born near the cut-off date of January first remain the relatively oldest in their respective year; however, the constituent year reflects the reality that the same player can be the younger within a two-year age band in one playing season but the older in the subsequent season.

**Figure 1:**
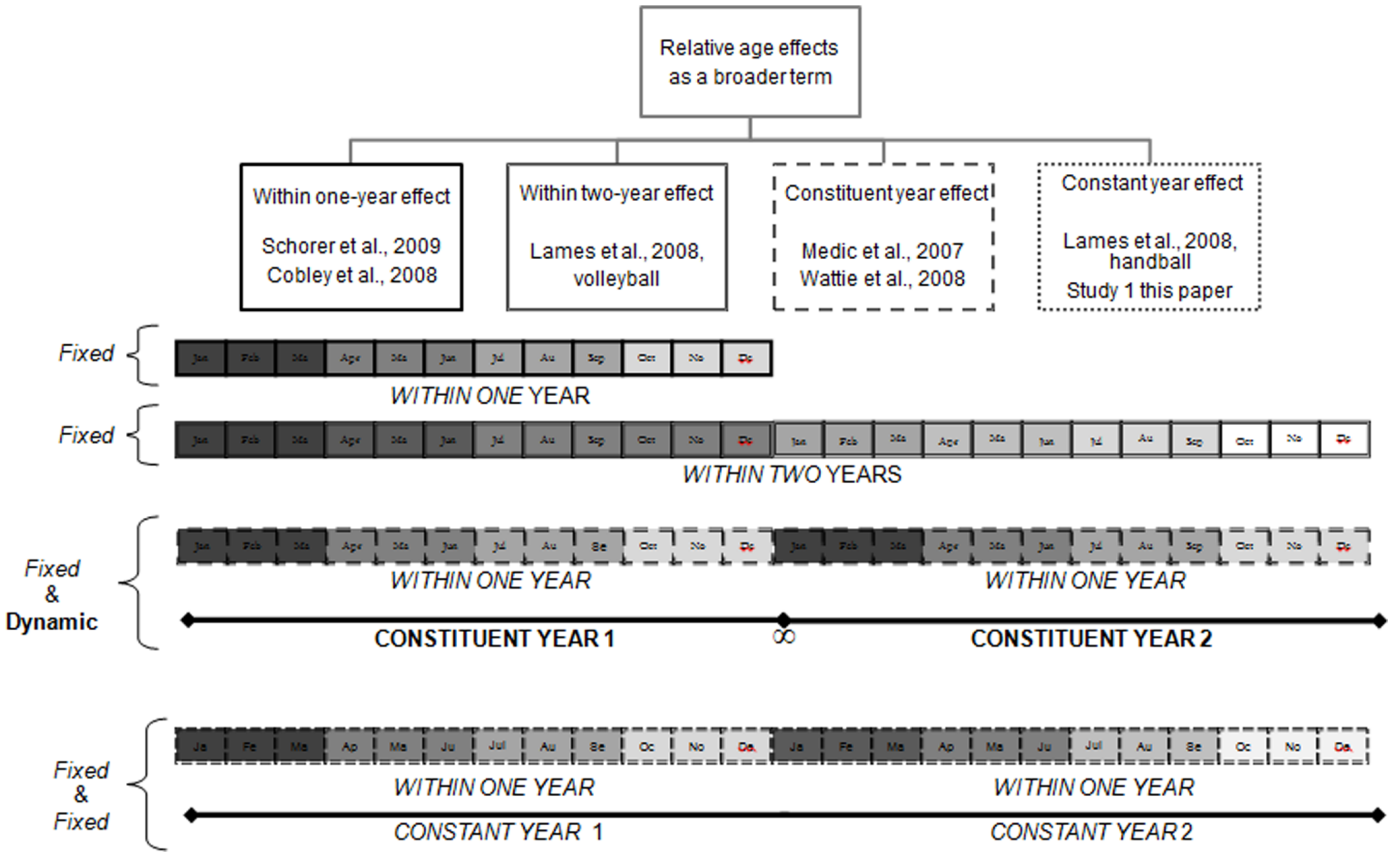
A system of relative age effects highlighting the independent influence of within-year effects, constituent year effects and constant year effects.

However, within the relative age literature, research has only considered relative age by itself, and has not looked at RAEs alongside other age grouping effects (i.e., constituent year effects) resulting from youth sport participation structures that may create demographic effects distinct from those previously described. Furthermore, research has seldom considered the impact of sport structures within age grouping systems that differ from the typical 12-month annual age grouping. Indeed, at least four similar but distinct age grouping systems can be identified. Figure 1 illustrates these different age grouping effects in sport, all under the umbrella of RAEs. For clarity, w*ithin one-year* and *within two-year effects* refer to traditional relative age effects, where the eligible selection cohort spans a 12 and 24 months range, respectively. In both *within one-year* and *within two-year* age grouping systems, relative age is a fixed characteristic (not changing from year-to-year). *Constituent year effects* refer to effects observed when multiple *within one-year* cohorts participate in multiyear age bands, such as in the 2-year bands in youth ice hockey [29], or the 5-year bands in masters sport [26]. As previously described, an individual's relative age at the *constituent year* level is a dynamic characteristic, changing from year-to-year. Finally, *constant year effects* are similar to *constituent year effects*, with the significant difference being that the multiyear groupings are kept constant (fixed) across development. In these systems, athletes are kept in this same age band (i.e, at Under 13 or Under 14), and this band moves as a of two year age group across the development system; meaning they move from Under 13 & Under 14 to Under 15 & Under 16, and so on.

The purpose of this investigation was to explore the aforementioned *constant year* age-grouping structure in sport that may create different effects on participation in elite sport. The *constant year* structure was one where a 12 month annual age grouping is in place, but where participation is simultaneously organized in two-year age bands (i.e., two 12 month annual age groups). In this system, participants have a ‘relative age’, but will also occupy a different position within the two-year age band (first vs. second constituent year). While these two-year constituent years can be dynamic, as was shown in a previous study [29], they can also be fixed during youth development [31]. The latter, fixed *constant year* structure is the target structure for the two studies presented here. The participation structures explored in Studies 1 and 2 highlight concomitant challenges to accurately and unambiguously define the terminology used to describe the consequences associated with different selection/de-selection policies.

## Introduction Study 1 - Constant year effects in national team players

**Figure 2:**
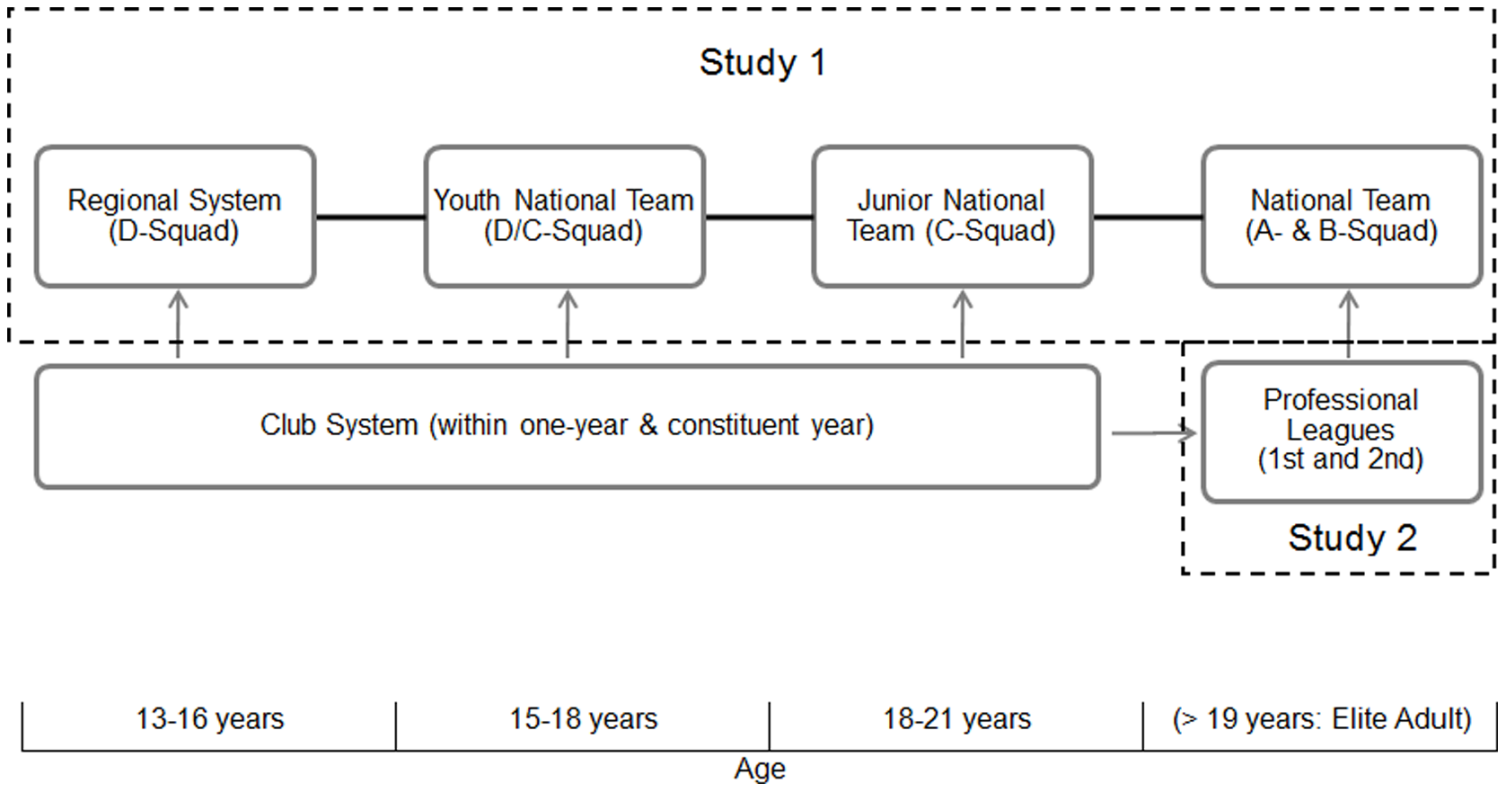
Talent development pathways in German handball.

In European handball, members of the German national teams are scouted in *within one-year* age bands each year (cf. Figure 2), but two adjacent *within one-year* bands are taken together to form the national team [32]. In contrast to where constituent years are dynamic (such as handball at the club level), here the relative position of year groups does not change over time (i.e., the youngest year group always remains the youngest of the two years being considered for the national team [31]. It should be noted that in German handball, those systems operate together. So players who are selected for the national talent pathway remain with their club, receiving additional training and support from the national coaches. The effects of such an age-structure for youth development have rarely been investigated [31]. This is different from typical constituent year effects where each person alternates between relative ages across youth development, at times being the relatively oldest while at other times being relatively youngest. In the constant year structure one can assume that the relatively older have an advantage for the complete duration of the talent development program. As for general relative age effects, the mechanisms described in the maturation hypothesis and selection hypothesis might cause similar differences in the distributions. Therefore, we hypothesized that the relatively older year groups in this type of system are advantaged and therefore there should be more players born in these year groups chosen for the national team.

The aim of this study was to examine the effects of a ‘constant year’ grouping in youth handball (i.e., a constant year effect) to supplement results from Schorer, et al. [1] showing within year effects for this sample. While the presentation of differences in distributions of relatively younger and older is generally sufficient, some of the strongest evidence for RAEs within annual age-groupings comes from studies showing shifts in the effects when cut-off dates are changed [33], [34]. Fortunately for our examination, this was done by the European Handball Federation for female players born between 1985 and 1987 when they created a ‘one time only’ three year age band (1985–87). As a result, instead of being the relatively oldest year group in a two year band of players born in 1987 and 1988, the 1987 year born players become the relatively youngest group in a team incorporating a three year band. Therefore, a clear shift should be observable in female handball corresponding to this unique perturbation, while there should be no shift for male players.

### Methods Study 1

The current study was a secondary analysis of data described by Schorer et al. [1], which had data for 3247 (53.4% female) athletes within the German Handball Federation between the 1993–2007 seasons. Within this dataset, there were five sub-categories, or levels, that describe an athlete's age and stage of participation within the German handball development system proceeding from a regional developmental system (i.e., D-squad, the lowest level) to the highest level, the National team (i.e., A-squad). Of the total sample size (*n = *3247) of this dataset, the regional developmental system was comprised of 734 athletes aged 12–16 years from more than 20 regional federations (D-squad; n_male_ = 401, 13–16 years; n_female_ = 333, 12–15 years). The next level represented the German youth national system, comprised of 1019 athletes aged 15–18 years (D/C-squad; n_male_ = 517, 16–18 years; n_female_ = 502, 15–17 years). The third level within the German handball development system was the junior national team (C-squad; n_male_ = 184, 19–21 years; n_female_ = 327, 18–20 years), respectively. Beyond the C-Squad level, starting at approximately of 19 years of age, players were no longer grouped by age, as in squads D, D/C and C, and could be selected to one of two teams. The highest of these teams was the adult National team (i.e., A squad), which participated in the Olympic Games and/or the World Championships. This dataset contained 670 A-squad athletes (n_male_ = 236; n_female_ = 434). Athletes who were not chosen for the A-squad National team could be chosen for the B-squad National team, which was viewed as a level to develop and support younger players who may someday reach the A-squad. This dataset contained 313 B-squad athletes (n_male_ = 175, n_female_ = 138).

To test for constant year effects, birth-dates for all players were analyzed. Their birth year was re-coded to reflect his or her birth year position into year 1 for the older athletes and year 2 for the younger athletes. As birth rates were not assumed to change significantly from one year to the next, we assumed an equal distribution between years and comparisons were based on the differences in percentages of players from year 1 compared to year 2. As noted above, an exception occurred for the female athletes born in 1987. Due to an international ruling, they were sorted into a ‘one time only’ triple year group of players born between 1985 and 1987. So for female players considered in this study born before 1985 the odd years were always the older ones, while for girls born in 1988 and after the even years were the older ones.

To test for differences between distributions between players from year 1 and year 2, Chi-square-tests were calculated with the effect size w reported [35]. SPSS 20.0 and G*Power 3.10 were used for all statistical analyses [36].

### Results Study 1

As can be seen in Figure 2, examinations of the male athletes showed that the older year groups (black columns) within each double year team were over-represented in comparison to the younger year groups in the D/C-squad (grey columns), χ^2^(1) = 29.26, *p*<01, *w* = 24, Y1: 61.9%. This pattern of results was also found for the C-squad, χ^2^(1) = 26.63, *p*<01, *w* = 38, Y1: 69.0%. The pattern diminished in the B-, χ^2^(1) = 3.02, *p* = 09, Y1: 43.4%, and A-squads, χ^2^(1) = 0.15, *p* = 75, Y1: 48.7%, where no consistent pattern was found.

**Figure 3:**
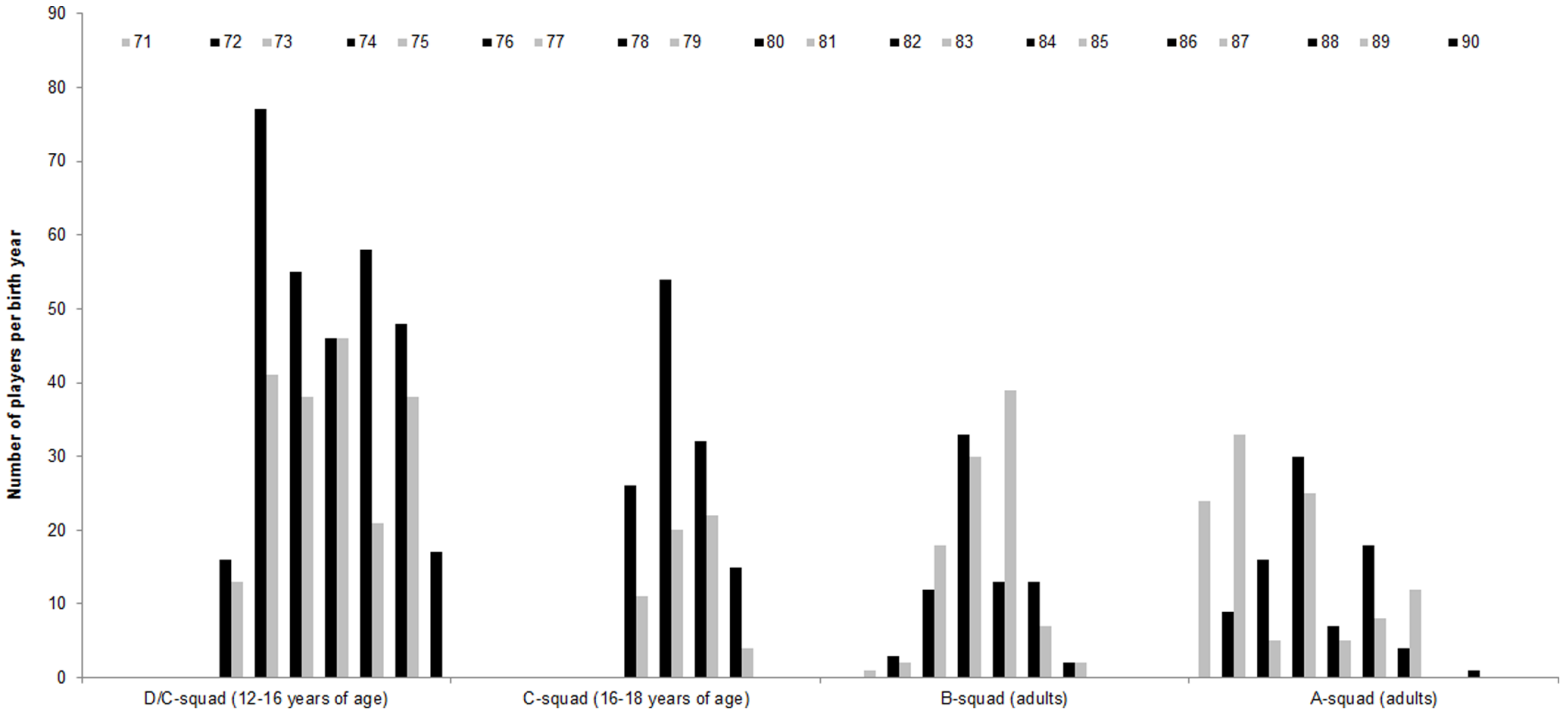
Constant year effect with number of male players per birth year across competitive levels in German handball showing an over representation of older (dark bars) in comparison to younger (light bars) male players in D/C and C levels but not in B and A levels.

For female athletes a similar pattern was observed (c.f., Figure 3). A significant over-representation was found for the D/C-squad, χ^2^(1) = 65.30, *p*<01, *w* = 37, Y1 = 68.7%, as well as for the C-squad, χ^2^(1) = 51.20, *p*<01, *w* = 40, Y1 = 70.2%. As expected, the change from older year groups being the odd years before 1985 to being from even years for players born in 1988 created a shift in distributions. Again, the same patterns were not observable for the open-age adult national teams, B-squad: χ^2^(1) = 1.04, *p* = 35, Y1 = 45.7%, A-squad: χ^2^(1) = 0.59, *p* = 48, Y1 = 51.8%.

### Discussion Study 1

The aim of this study was to explore the concept of a constant year effect in youth handball and to investigate whether a change in year groupings in female handball would cause a shift in patterns similar to those found in within year effects in soccer [33], [34]. As hypothesized, there were constant year effects in the D/C-squads and C-squads for boys and girls. The effect sizes generally show small to moderate effects (range.24 to.40) for these birth year groupings [35]. Perhaps the more convincing evidence comes from the shift in birth year groupings in the female players born between 1985 and 1987. Here a clear shift was found indicating these are not random effects, but ones that are driven by the birth year grouping strategy. Interestingly, an over-representation of females born in 1987 compared to those born in 1986 was found in the data. At the time this decision was made, actors in the youth development system in Germany feared that the 1987 birth year group would be lost because they had to compete for spots with two older year groups. This lead the German Handball Federation to implement strategies to ensure that the best players across these three years would stay within their talent development system, such as increasing the numbers of spots especially for females born in 1987.

This effect diminished for both sexes at the adult national team levels (B- and A-squad). There are two possible explanations. First, it is possible that as athletes progress through the talent development pathway the effect diminishes, a relationship seen for within year effects [19]. However, if this was the case a descriptive trend should still be observable, which did not occur in these data. Therefore, it seems more plausible to assume that the effect vanishes because there are no birth year groupings in adult handball (i.e., it is an open-age team). There seems to be no carry-over effect from youth handball for a highly selected team like the national teams. This might be best explained by the fact that a national team normally consists of 14 players from an age range of approximately 15 years (Age: 20–35 years). So from each birth year, in general between zero and four players are invited to join the national team. However, carry-over effects may still be possible in less elite samples, which we investigate in study 2.

## Introduction Study 2 - Distribution of Birth Year in Professional Male Handball in Germany

While Study 1 demonstrated *constant year effects* at the younger developmental levels (D-squad: 12–16 year olds; C-squad: 15–18 year olds), to be able to estimate the implications of the constant year effect, it is important to understand the stability of the effect across development and in different developmental pathways. For example, previous research has shown that the magnitude of *within one-year* RAEs can change significantly between youth and elite adult levels of play [19]. In ice hockey approximately 40% of players are born in the first three months of the selection year (Jan-Mar) at the youth [2] and amateur developmental leagues [3], while among professionals approximately 30% of players are born during this same period [3], [30]. Similarly, a study of 1990 World Cup players found that 45% of players on competitive youth football (soccer) teams (under-17s) were born in the first three months of the selection year, a proportion that decreased to 28% among senior national teams [37]. While these trends are not completely understood, they nevertheless indicate the variable salience of an age grouping structure's influence on talent development at different stages of athlete development.

**Figure 4:**
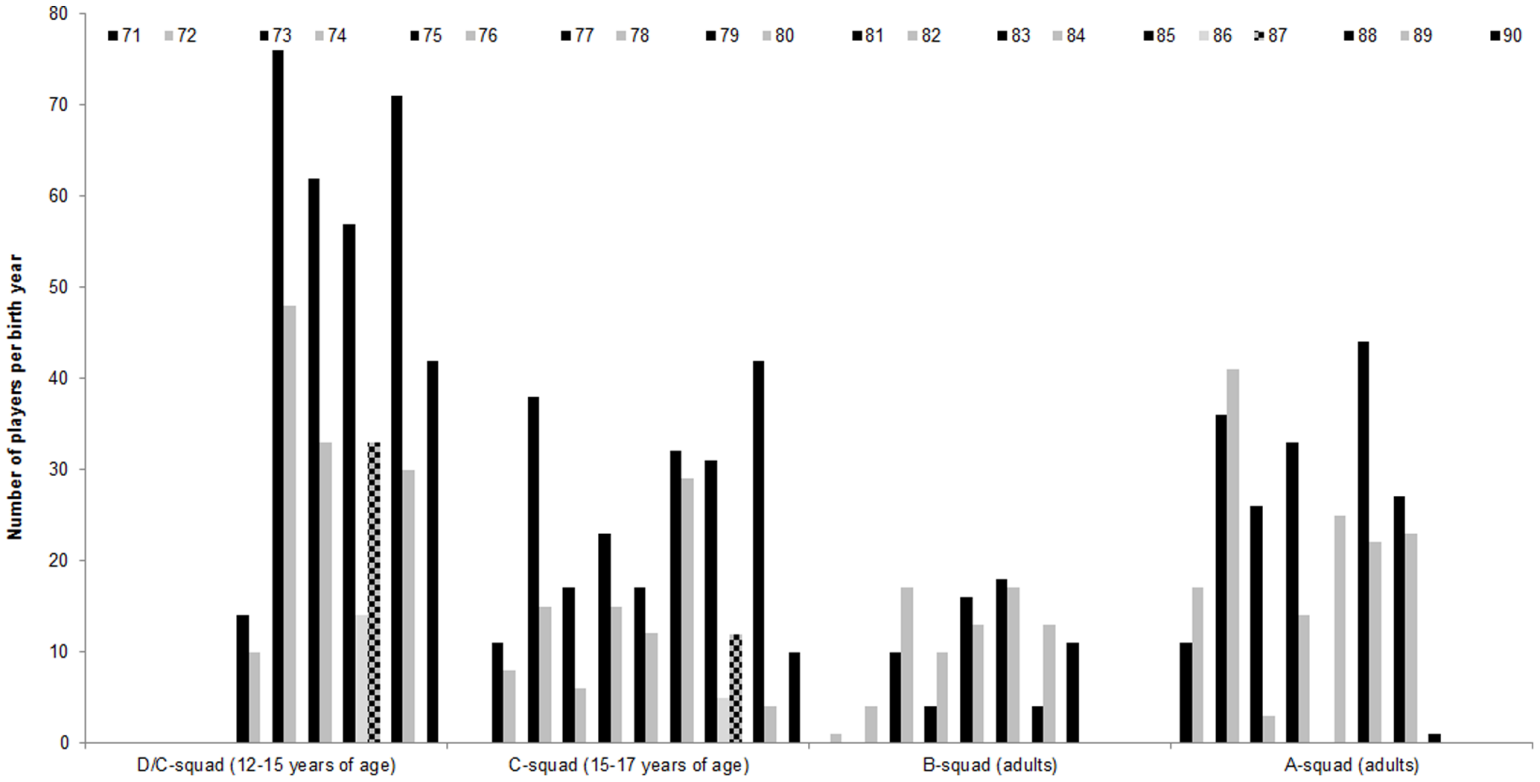
Constant year effect with number of female players per birth year across competitive levels in German handball showing an over representation of older (dark bars) in comparison to younger (light bars) female players in D/C and C levels but not in B and A levels. Note: Change in double years for players born 1985 to 1987 (checkered bars)

German handball is an interesting sport to investigate this issue, because young athletes develop in two parallel systems (see Figure 4), the club system and the national talent development path [32]. In the club system, athletes train multiple times a week while also meeting the requirements of the national talent development path. As seen in Study 1, the national talent development path leads to a constant year effect in the D/C and C-squad, because two one-year groups are taken together. Conversely, the club development has a constituent system, where athletes rotate from being the older group one year to the being the younger the next (www.dhb.de). Of particular relevance to the current study, Schorer and colleagues [1] found mixed results with respect to *within one-year* RAEs in German handball. While no *within one-year* effects were found for the adult national teams, there were some for the first and second league of *professional* handball. Interestingly, Study 1 findings suggest that parallel trends exist with respect to *constant year effect*s among adult national team athletes. However, to determine whether *constant year* effects completely parallel *within one-year* effects, there is a need to determine whether effects from the national talent development path carry over to the elite *professional* leagues. Therefore, the aim of this study was to investigate *constant year effects* in adult professional handball in the highest two leagues in Germany.

### Methods Study 2

The German Handball League provided data for 2291 male players from the first league and 4824 from the two second leagues identified from the 1998/1999–2005/2006 seasons. The German Handball Leagues are organized hierarchically, with the first league as the most elite one, in which the teams play for the German Championship, followed by the second league, and so on. The players in the first two male leagues are professionals. Of these 7115 elite athletes, 5326 players were German (i.e., national) and 1789 were from outside Germany (i.e., international). Because players with birth years prior to 1963 had less than 50 cases per birth year, we excluded these observations from our analyses ( = 1.6% of all cases). For two of the athletes the birth year was missing and they were also excluded from analyses. As in Study 1, each player's birth year was re-coded to reflect his birth year position into year 1 (for the older players) or year 2 (for the younger players). As with Study One, an equal distribution between years was assumed and analyses compared percentages of players from year 1 compared to year 2 using Chi-square-tests. Effect size (*w)* and test power (1 – β) were also reported [35]. SPSS 20.0 and G*Power 3.10 were used for all statistical analyses [36].

### Results Study 2

**Figure 5:**
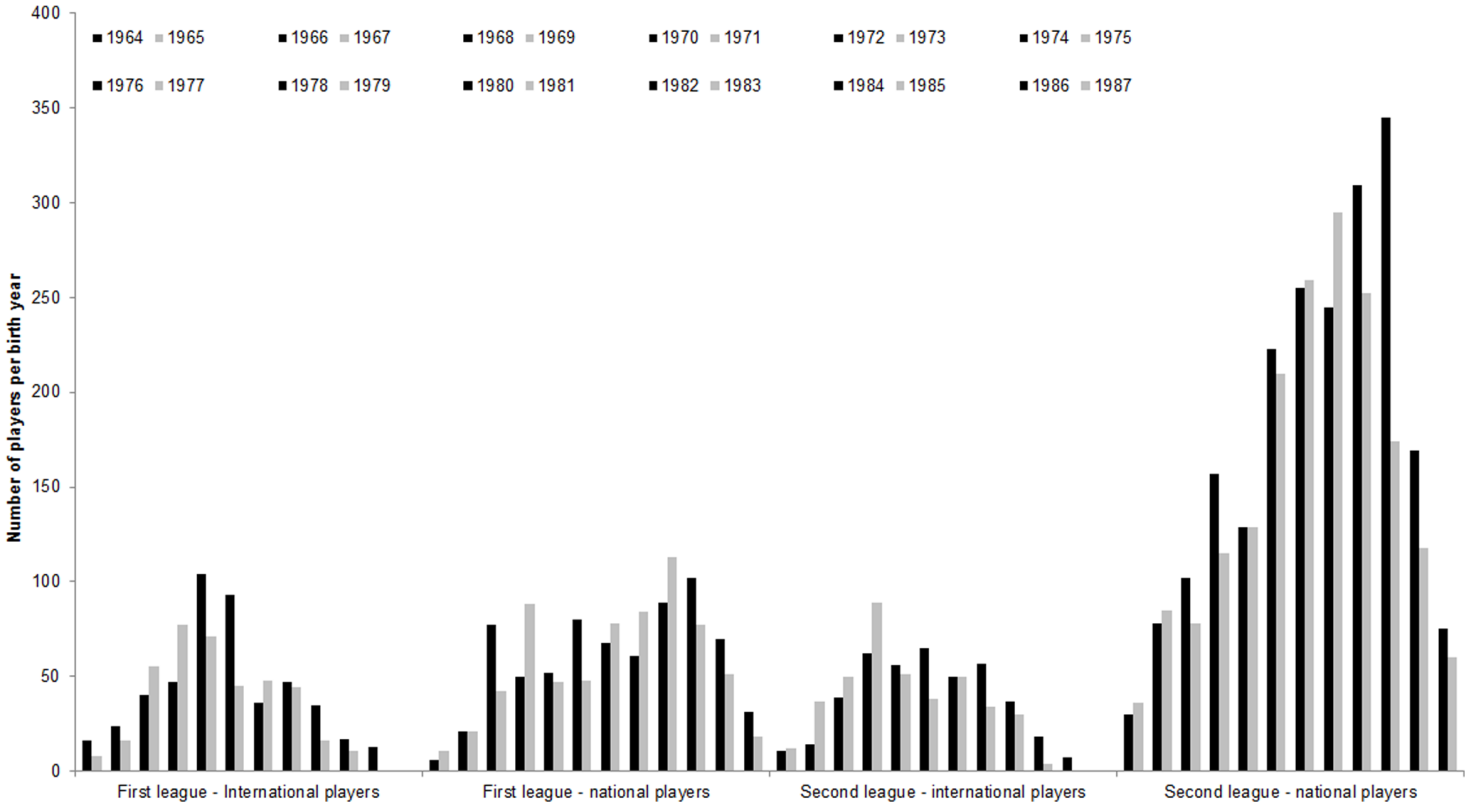
Birth year distributions in 1st and 2nd leagues of German handball for national and international male players differentiated by older (dark bars) and younger (light bars) players.

Fifty-three percent of all players were born in the older year, resulting in a small but significant difference in birth year patterns, χ^2^(1, *n* = 6999) = 26.54, *p*<01, *w* = 06. Differentiating between leagues, the older players were overrepresented in the first, 52.3%, χ^2^(1, *n* = 2253) = 4.89, *p* = 03, *w* = 05 and the second league, 53.4%, χ^2^(1, *n* = 4746) = 22.39, *p*<01, *w* = 07. When considering national and international players separately, only the birth year patterns for the international players in the first league, 54.4%, χ^2^(1, *n* = 868) = 6.65, *p* = 01, *w* = 09, and the national players in the second league, 53.9%, χ^2^(1, *n* = 3928) = 23.84, *p*<01, *w* = 08, reached significance. For the national players in first league, 51.0%, χ^2^(1, *n* = 1385) = 0.61, *p* = 45, *w* = 02, 1–β = 11, and the international players in second league, 51.2%, χ^2^(1, *n* = 818) = 0.49, *p* = 51, *w* = 02, 1–β = 10, there were no significant differences between older and younger birth years(cf. Figure 5).

### Discussion Study 2

Study two explored whether the constant year effect noted in the national talent development system remained in the professional adult handball leagues or whether the club development system with constituent birth years would be stronger in its effect. At first glance, the significant differences between older and younger birth years support an effect of the constant year grouping by the German national talent development pathway on this elite sample. However, the observed effect sizes are quite small suggesting the practical relevance of these results is limited [35]. So instead of supporting our alternative hypothesis of an effect of the constant year grouping in the national talent pathway, these results may provide stronger support for the null-hypothesis. In our case this would mean that the club system plays a stronger role in the development of future professional handball players, because there a constituent year system is used which would theoretically confer no advantage over time. This stronger effect might be best explained by amount of training; handball clubs train with their youth players multiple times a week, while the national team only meets once every two to three months for structured training camps.

Almost the same line of argumentation can be used for the international and national players in the first and second league. While we found an interesting pattern of significant results - the first league national players and the second league international players demonstrated significant differences while the other two combinations did not – these should not be over-interpreted. Again, the effect sizes are rather small and limit the practical relevance of these effects.

## General Discussion

Moving forward with research in this area, it will be important to understand the strength of each respective effect across development. For example, Schorer, et al. [1] noted that effect sizes of within year effects decreased for the male and female handball players (from *w* = 39 to.32 for the males and from *w* = 24 to.17 for the females) from the D/C-squad to the C-squad (i.e. from 15 to 21 years of age), while the current results showed an increase in effect sizes for the constant year effect from *w* = 24 to.39 in the males and from *w* = 37 to.40 in female players (study 1). While this must be cautiously interpreted since this is the first examination of this phenomenon, it seems there are different processes happening across the age groups. The strongest effect for within year effects happened during the first selection level at the D/C-squad and then decreased before eventually vanishing at the adult level [1]. Conversely, the constant year effect increased from the D/C-squad to C-squads. Interestingly both effects vanish in the adult levels in the B- and A-squads. This might be explained by an informal strategy used within the national talent development system. While during the D-/C-squads both year groups are taken together and the younger ones are given a chance to rise up to the level of the older year groups, in the following squad mostly performance measures are taken into account irrespective of the year group the players are in. The competition for spots could then lead to a stronger effect for year groups in the C-squad as it was in the previous D-/C-squad. But this hypothesis requires verification.

It is also important to understand the cumulative or multiplicative influence of these effects since they likely interact in varying ways across development. Based on the evidence from these studies and prior work on within year effects, it seems it would be particularly disadvantageous to be in the youngest quartile in a one year age group (a within one-year effect) and born in the earlier of two years in a two year constant age band (a constant year effect). Although these relationships are difficult to test inferentially due to limitations in analysis of interactions in non-parametric statistics, a more comprehensive understanding of these factors (and the mechanisms perpetuating their effects) would be helpful for models of long-term athlete development.

In summary, these results highlight a new phenomenon in research on the relative age effect in sport and emphasize the need for an encompassing model of the varying social policy implications across athlete development. Moreover, it is important to understand the different constraints that are imposed on athlete development from different levels of sport (e.g., the club versus the national talent development systems) and across different sports (e.g., between German handball to Canadian ice-hockey). As shown in this study, the unique policy and administrative constraints of the German handball system have resulted in specific age-related biases in athlete development outcomes. These results, coupled with the work of other researchers in this area, suggest caution should be taken in future studies to adequately describe the range of variables interacting and constraining athlete development.
