# Correction: A New Dimension to Relative Age Effects: Constant Year Effects in German Youth Handball

**DOI:** 10.1371/annotation/346754d7-87e5-4e5f-90c8-86a23632227a

**Published:** 2013-05-06

**Authors:** Jörg Schorer, Nick Wattie, Joseph R. Baker

The affiliations for Dr. Jörg Schorer are incorrect. The full, correct author byline and affiliations are as follows:

Jörg Schorer^1,3^, Nick Wattie^2^, Joseph R. Baker^2^

**1** Institute of Sport and Exercise Sciences, University of Muenster, Münster, Germany, **2** School of Kinesiology and Health Science, York University, Toronto, Canada, **3** Institute for Sport Science, University of Oldenburg, Oldenburg, Germany

